# Defining the Influence of the A12.2 Subunit on Transcription Elongation and Termination by RNA Polymerase I In Vivo

**DOI:** 10.3390/genes12121939

**Published:** 2021-11-30

**Authors:** Andrew M. Clarke, Abigail K. Huffines, Yvonne J. K. Edwards, Chad M. Petit, David A. Schneider

**Affiliations:** Department of Biochemistry and Molecular Genetics, University of Alabama at Birmingham, Birmingham, AL 35294, USA; andrew.martin.clarke@gmail.com (A.M.C.); mcconaha@uab.edu (A.K.H.); yedwards@uab.edu (Y.J.K.E.); cpetit@uab.edu (C.M.P.)

**Keywords:** RNA polymerase I, RNA synthesis, rRNA, transcription termination, transcription elongation, NET-seq

## Abstract

*Saccharomyces cerevisiae* has approximately 200 copies of the 35S rDNA gene, arranged tandemly on chromosome XII. This gene is transcribed by RNA polymerase I (Pol I) and the 35S rRNA transcript is processed to produce three of the four rRNAs required for ribosome biogenesis. An intergenic spacer (IGS) separates each copy of the 35S gene and contains the 5S rDNA gene, the origin of DNA replication, and the promoter for the adjacent 35S gene. Pol I is a 14-subunit enzyme responsible for the majority of rRNA synthesis, thereby sustaining normal cellular function and growth. The A12.2 subunit of Pol I plays a crucial role in cleavage, termination, and nucleotide addition during transcription. Deletion of this subunit causes alteration of nucleotide addition kinetics and read-through of transcription termination sites. To interrogate both of these phenomena, we performed native elongating transcript sequencing (NET-seq) with an *rpa12Δ* strain of *S. cerevisiae* and evaluated the resultant change in Pol I occupancy across the 35S gene and the IGS. Compared to wild-type (WT), we observed template sequence-specific changes in Pol I occupancy throughout the 35S gene. We also observed *rpa12Δ* Pol I occupancy downstream of both termination sites and throughout most of the IGS, including the 5S gene. Relative occupancy of *rpa12Δ* Pol I increased upstream of the promoter-proximal Reb1 binding site and dropped significantly downstream, implicating this site as a third terminator for Pol I transcription. Collectively, these high-resolution results indicate that the A12.2 subunit of Pol I plays an important role in transcription elongation and termination.

## 1. Introduction

**Figure 1:**
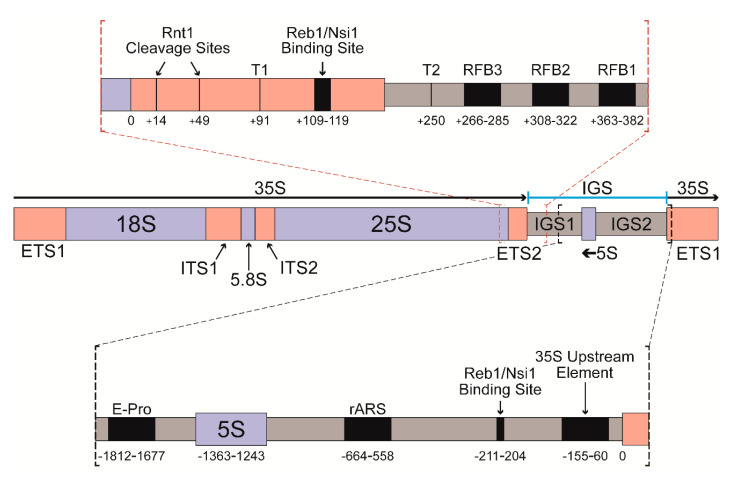
Gene diagram of an *S. cerevisiae* rDNA repeat. Black arrows indicate the direction of transcription; red bracketed insert details the portion of the rDNA containing the two sites of Pol I termination as well as the RFB region. The indicated coordinates are relative to the mature end of the 25S rRNA. The black bracketed insert details the portion of the rDNA containing the E-pro, 5S gene, and IGS 2. The indicated coordinates are relative to the transcription start site of the downstream 35S repeat.

Ribosome biogenesis in *S. cerevisiae* (yeast) begins with transcription of the 35S gene by RNA polymerase I (Pol I) to synthesize the 35S ribosomal RNA (rRNA). The 35S rRNA is then co- and post-transcriptionally cleaved to produce the 18S, 5.8S, and 25S rRNAs (Figure 1). These rRNAs, along with the 5S rRNA synthesized by RNA polymerase III, form the RNA backbone of ribosomes. Rapidly dividing yeast cells have a tremendous demand for ribosomes. This is reflected by the fact that more than 60% of cellular transcription is devoted to rRNA synthesis [1].

Yeast have approximately 200 copies of the 35S gene to accommodate this demand, arranged tandemly on chromosome XII [1]. Half of these copies are transcriptionally active, with each actively transcribed copy being host to approximately 50 Pol I transcription elongation complexes (ECs) [2]. In between each rDNA copy is an intergenic spacer (IGS) consisting of the 5S gene flanked by intergenic spacers 1 and 2 (IGS 1 and IGS 2). As shown in Figure 1, the IGS 1 and IGS 2 regions contain many features that are important for transcription termination as well as the reduction in collisions between transcription and replication machineries. 

Early studies identified two sites of Pol I transcription termination [3]. The first site (referred to as T1) is 91 base pairs downstream of the mature end of the 25S rRNA and represents the main site of Pol I transcription termination [4]. T1 lies at the end of a 20 bp tract of A/T-rich rDNA. The binding site for the yeast DNA binding protein Reb1 is located downstream of this A/T-rich sequence. In combination with Reb1, these two sequence elements are sufficient to terminate Pol I transcription in vitro [5]. An early model proposed that the mechanism of termination by Pol I consisted of a Reb1-mediated “pause” followed by “release” of the nascent transcript due to the A-rich template DNA tract [6]. Previous studies identified that murine transcription termination factor I (mTTF-I) could also terminate yeast Pol I transcription in vitro [7]. Extensive sequence similarity between mTTF-I and Reb1 supports this mechanism and establish the possibility of a universally conserved mechanism of Pol I transcription termination in all eukaryotes [8]. However, the mechanism of Pol I termination in yeast is likely much more complicated than these original models suggest. Some recent studies have cast doubt on Reb1′s role in yeast Pol I termination [9,10], while others have implicated a host of new factors in Pol I termination including RNase III Rnt1 [4] and 5′ exonuclease Rat1 [11]. Additionally, a Reb1 homolog, Nsi1, was found to be required for efficient Pol I termination in vivo [10], in contrast to Reb1 [9]. 

Interestingly, Reb1 is also implicated in the initiation and termination of transcription by Pol II in yeast. Reb1 binding at sites within a subset of Pol II promoters is required for the formation of nucleosome-free regions (NFRs), thereby allowing transcription of those genes [12,13]. Reb1 binding to sites downstream of Pol II genes has also been shown to pause Pol II transcription in vivo in a polar manner, prompting ubiquitination and subsequent Pol II transcription termination [14], similar to its originally suggested role in Pol I transcription. 

The current model for Pol I termination at T1 is similar to the “torpedo” model for Pol II termination [9,10]. Pol I transcribes to position +91 downstream of the mature end of the 25S rRNA and halts upstream of the binding site for Nsi1. Concurrently, Rnt1 recognizes a stem-loop formed in the nascent RNA transcript just downstream of the 25S end, and cleaves between positions +14/+15 and positions +49/+50 [15]. This cleavage produces a 5′ monophosphate terminus on the nascent transcript, which is the appropriate substrate for the Rat1 exonuclease [16]. As in Pol II termination, Rat1 is proposed to be the “release factor” for Pol I, so that termination occurs when Rat1 reaches the stalled Pol I EC [11]. However, no mechanism has been put forward describing how this release occurs. Termination at the “failsafe” site downstream of T1 (known as T2) is less well-studied, although it has been shown to involve RFB region binding proteins Fob1 and Rat1 [11]. Previous work also suggests an additional potential termination site, Tp, adjacent to the promoter-proximal binding site for Reb1 [3,17]. 

One of the known Pol I subunits involved in both intrinsic RNA cleavage [18] and transcription termination [19] is Rpa12 (A12.2). This subunit is homologous to Rpb9 in Pol II and Rpc11 in Pol III. The C-terminal domain of A12.2 also bears homology to Pol II cleavage factor TFIIS [20]. *rpa12Δ* strains grow very slowly compared to wild-type (WT) at 30 °C [21] and are deficient in Pol I transcription termination, demonstrated by Miller chromatin spreads [19]. Structural studies of the interaction between the Reb1 and Pol I ECs in *Schizosaccharomyces pombe* revealed that protein-protein interactions between Reb1 and the A12.2 subunit are specifically required for transcriptional arrest and termination of Pol I ECs [22]. This observation may explain the previously identified orientation dependence in Reb1 transcriptional arrest activity with respect to *S. pombe* Pol I [23] and *S. cerevisiae* Pol II [14]. In addition to its roles in cleavage and termination, recent work from our lab demonstrated that A12.2 also affects the stability of the Pol I EC [24] and is directly involved in nucleotide addition by Pol I [24,25,26]. 

Many biochemical and structural analyses have implicated A12.2 in a myriad of activities. Here, we use native elongating transcript sequencing (NET-seq) to investigate how deletion of this subunit influences Pol I activity in vivo. This technique allows us to reproducibly determine Pol I occupancy with single-nucleotide resolution, providing detailed insight into Pol I transcription elongation properties in living cells [27,28,29]. Upon deletion of *RPA12*, we observed re-ordering of median polymerase occupancy throughout the 35S gene, favoring the 5′ end of the template. Furthermore, we identified robust changes in Pol I occupancy in response to the rDNA template sequence between the WT and *rpa12Δ* strains, confirming that A12.2 affects Pol I nucleotide addition in vivo. Our experiments also demonstrated that the *rpa12Δ* strain is termination deficient in vivo, with significant *rpa12Δ* Pol I occupancy observed throughout the IGS. Strikingly, *rpa12Δ* Pol I occupancy increases dramatically directly upstream of the promoter-proximal Reb1 binding site, indicating that the *rpa12Δ* Pol I EC is paused or halted stably at this site. These findings provide insight into A12.2′s role in growing cells and pose intriguing new questions about Pol I termination and the conserved genomic architecture of the IGS. 

## 2. Materials and Methods

### 2.1. Native Elongating Transcript Sequencing (NET-seq) Library Preparation and Sequencing

NET-seq library generation was performed exactly as previously described using the original HA-tagged Rpa135 *S. cerevisiae* strain [27] and a derivative of this strain carrying a full deletion of *RPA12*. In brief, early log-phase cultures were collected via filtration, then flash-frozen in liquid nitrogen and cryogenically lysed by a mixer mill. Pol I elongation complexes were isolated via immunoprecipitation with anti-HA magnetic beads. Associated RNA transcripts were isolated using an acidic phenol-chloroform extraction. The RNA transcripts were 3′ appended with a DNA linker and reverse transcribed to produce complementary DNAs (cDNAs). The cDNAs were circularized and amplified by PCR to produce high-throughput sequencing libraries. Finally, these libraries were sequenced as previously described [27].

### 2.2. NET-seq Data Formatting and Analysis

Sequencing trimming, alignment, and data formatting were performed as previously described [27]. Unix scripts are available upon request. Data can be accessed via the Gene Expression Omnibus database with the accession number GSE142457.

**Figure 2:**
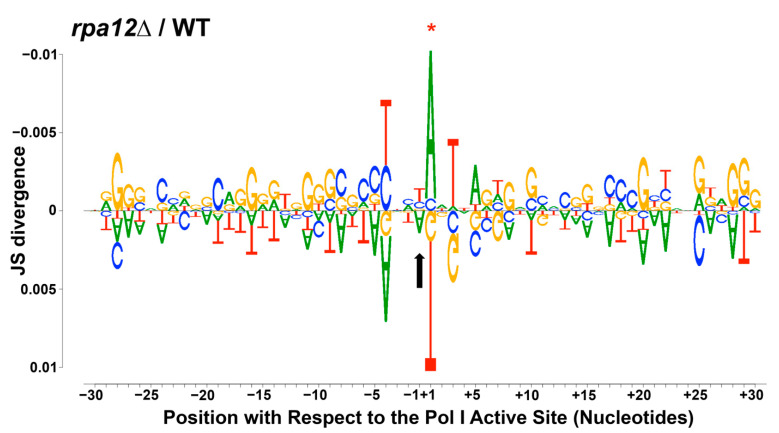
*RPA12* deletion alters the relationship between Pol I occupancy and nucleotide enrichment in the RNA:DNA hybrid and the template sequence both up- and downstream of the polymerase. A DiffLogo was generated to visualize template sequence enrichments at the top 2.5% occupied positions by Pol I for the *rpa12Δ* strain (above the 0 axis) and the WT strain (below the axis). The black arrow indicates the last incorporated nucleotide (LNT), as this graph is oriented with respect to the polymerase. The asterisk denotes a significant difference between WT and the *rpa12Δ* strains at that position.

Data analyses methods in R (version 4.0.2) were based on previous literature [28]. In brief, the three replicates for the WT and *rpa12Δ* yeast strains were aggregated. To generate Figures, the counts were normalized to the sum of all signals on the positive strand of the 35S gene (corresponding to negative strand RNA transcripts). Analyses were performed using the following packages: R (version 4.0.2) [30], dplyr (version 1.0.2) [31], plyr (version 1.8.6) [32], ggplot2 (version 3.3.2) [33], ggseqlogo (version 0.1) [34], ggpubr (version 0.2.5) [35], cowplot (version 1.1.1) [36], matrixStats (version 0.58.0) [37], hexbin (version 1.28.1), tweedie (version 2.3.3), statmod (version 1.4.35), magritter (version 1.5), tidyr (version 1.1.2), seqinr (version 3.6-1), zoo (version 1.8-8), and scales (version. 1.1.1) [38]. The DiffLogo software package (version 2.14.0) [39] was used to generate Figure 2. R scripts are available upon request.

## 3. Results

### 3.1. WT and rpa12Δ NET-seq Libraries Are Highly Reproducible

**Figure 3:**
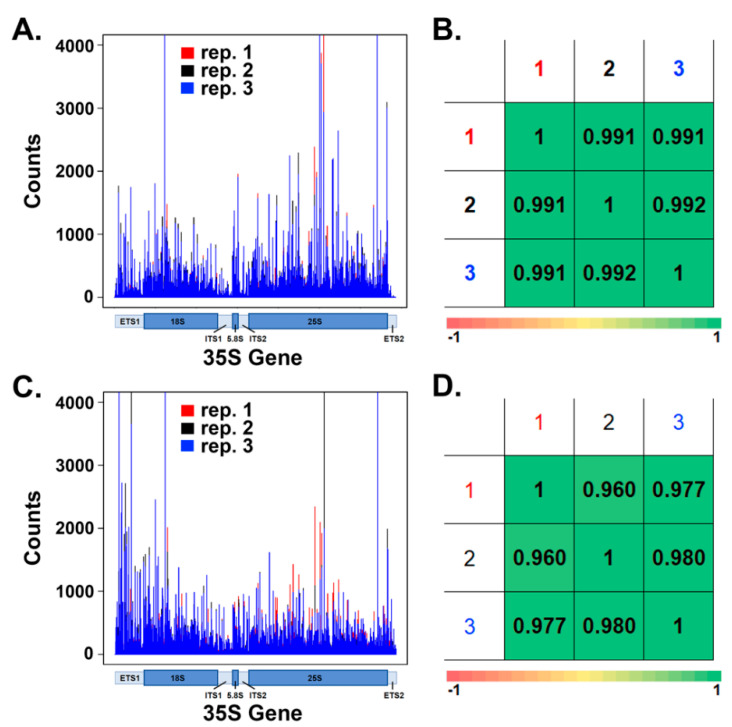
NET-seq experiments are reproducible. NET-seq was performed in biological triplicate and resultant reads corresponding to the 35S rDNA gene were isolated. A histogram for WT (**A**) and *rpa12Δ* (**C**) was generated, indicating Pol I occupancy at each position across the rDNA. The Spearman correlation test was executed to generate similarity coefficient values for WT (**B**) and *rpa12Δ* (**D**) libraries.

NET-seq was first developed to investigate the occupancy of Pol II at single-nucleotide resolution in vivo [40]. We recently optimized this technique to map Pol I occupancy [27,28,29]. To establish NET-seq as a reliable tool to investigate Pol I occupancy in vivo, we first determined if the results were reproducible. Therefore, we performed NET-seq in triplicate on WT and *rpa12Δ* yeast strains bearing HA-tagged Pol I as previously described [27]. The resultant reads were mapped to the 35S gene (Figure 3A for WT and 2C for *rpa12Δ*). The amplitude at each position corresponds to the number of polymerases mapped to that position, representing a snapshot of Pol I occupancy. The counts were normalized to the sum of Pol I occupancy from the 35S transcription start site (position 1) to the first termination site (position 6739). To determine the reproducibility among replicates of the same strain, the Spearman correlation test was deployed (Figure 3B for WT and 3D for *rpa12Δ*). This test ranks the peaks displayed in Figure 3A,C from highest to lowest for two replicates, compares those rankings, and generates a resultant coefficient value. This value indicates correlation similarity between replicates, where 1 represents 100% similarity. Therefore, values that are very close to 1 demonstrate that replicates display very similar occupancy patterns. As indicated in Figure 3B,D, the coefficient values generated for the WT and *rpa12Δ* strains indicated high reproducibility. These results reveal that NET-seq is an ideal tool to determine the effect of the deletion of A12.2 on Pol I occupancy in vivo.

### 3.2. RPA12 Deletion Alters Pol I Occupancy throughout the 35S Gene

**Figure 4:**
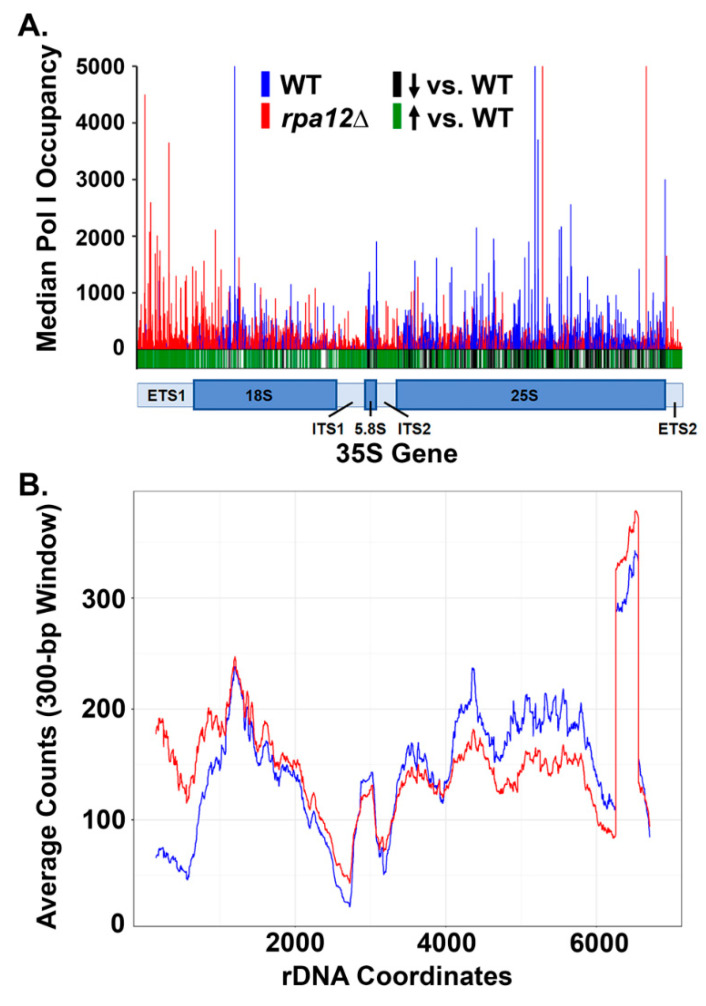
*RPA12* deletion shifts Pol I occupancy toward the 5′ end of the 35S gene. (**A**) Pol I median 5′ read end densities for WT (blue) and *rpa12Δ* (red) strains in three replicates each. Green lines indicate statistically significant increases in occupancy in *rpa12Δ* strain compared to WT. Black lines indicate statistically significant decreases in occupancy. Statistical analysis was performed using Student’s *t*-test (*n* = 3, *p*-value < 0.05). The 35S gene diagram is color coded for gene (dark blue) and spacer (light blue) regions. Occupancies are normalized to the sum of 35S occupancy. (**B**) The moving average across 300 bp of the median Pol I occupancy on the 35S gene for WT (in blue) and *rpa12Δ* (in red).

We demonstrated that NET-seq performed within yeast strains is highly reproducible, therefore, we can use this technique to draw conclusions about the occupancy of Pol I in the WT vs. *rpa12Δ* strains. The median occupancy for WT and *rpa12Δ* were plotted in Figure 4A, and a *t*-test was performed at each position to determine whether there was a significant change between strains. Based on the *p*-value, we determined whether there was a significant difference in occupancy and indicated this with either a green (increased occupancy) or a black (decreased occupancy) line below the histogram for the *rpa12Δ* strain as compared to WT. These results reveal that there is an increase in Pol I occupancy at the 5′ end of the rDNA in the *rpa12Δ* strain as compared to WT and indicates that the deletion of *A12.2* results in a significantly different occupancy pattern of Pol I across the template. Furthermore, there is an increase in Pol I occupancy in the ETS 2 region in *rpa12Δ* yeast. To examine these patterns further, the moving average across 300 nucleotides was plotted for the median occupancy (Figure 4B). This graph corroborates the findings from Figure 4A and shows that there is an increased Pol I occupancy at the 5′ and 3′ ends of the gene in the *rpa12Δ* strain. The simplest interpretation of these data is that loss of *A12.2* impairs processivity and alters positioning of Pol I, and the observed increase in occupancy at the 3′ end in the ETS 2 region is due to terminator read-through in the *rpa12Δ* strain (discussed below). It is reasonable to expect that there would be an increase in Pol I backtracking in the *rpa12Δ* strain, though NET-seq cannot be used to detect this effect.

### 3.3. Changes in Pol I Occupancy Are Template Sequence Dependent 

The results shown in Figure 4 demonstrate a 5′ bias in Pol I occupancy in the *rpa12Δ* strain, suggesting defects in transcription elongation. This finding is consistent with the results of transcription run-on experiments performed by Prescott, et al. [19] and previously published in vitro analyses of *rpa12Δ* Pol I by our lab [25]. However, neither study identified the underlying cause of the observed 5′ bias in transcription signal or reduced rate of nucleotide addition. Our lab recently determined that local nucleotide sequence affects nucleotide addition by Pol I in vitro and Pol I occupancy in vivo [27,29]. As a result, we tested whether the observed 5′ Pol I occupancy bias and overall occupancy differences observed in the *rpa12Δ* strain were sequence-dependent. We generated a DiffLogo (Figure 2) to visualize sequence enrichments for the top 2.5% occupied positions in the *rpa12Δ* strain (shown on top) vs. WT (shown below). The patterns shown in Figure 2 demonstrate that the deletion of *RPA12* repositions the polymerases on the rDNA template with respect to WT. In the *rpa12Δ* strain, Pol I is paused up- and downstream of G/C-rich rDNA regions, and there is a difference in sequence enrichment directly surrounding the LNT in the RNA:DNA hybrid as compared to WT. These results confirm that the *RPA12* deletion perturbs nucleotide addition by Pol I and demonstrate that the effects of this deletion are at least partially sequence dependent. This observation further supports the intimate relationship between template sequence and Pol I occupancy in vivo [27,29]. 

### 3.4. Deletion of RPA12 Results in Pol I Occupancy Downstream of T1 and T2

**Figure 5:**
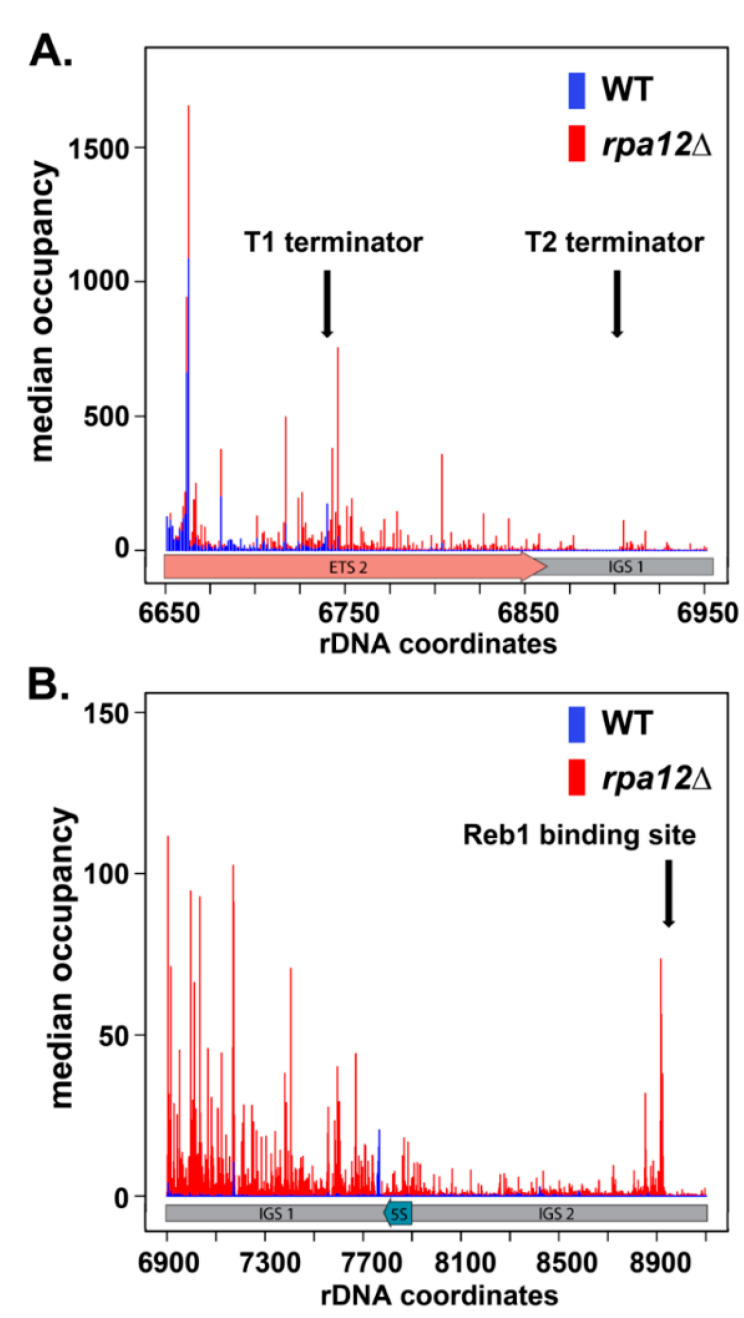
*RPA12* deletion reveals a putative third Pol I transcription termination site in the IGS. (**A**) Pol I median occupancy for WT (blue) and *rpa12Δ* (red) strains in ETS 2 and IGS 1 for three replicates each. Termination sites are highlighted with black arrows. Gene diagram is color coded for ETS 2 (salmon) and IGS 1 (grey). Occupancy is normalized to the sum of 35S occupancy. (**B**) Pol I median occupancy for WT and *rpa12Δ* strains in the IGS. The promoter-proximal Reb1 binding site is highlighted with a black arrow. Gene diagram is color coded for IGSs 1 and 2 (grey) and the 5S gene (cyan). The occupancy is normalized to the sum of 35S occupancy.

We observed a significant increase in the occupancy of Pol I in the ETS 2 region after *A12.2* deletion (Figure 4), suggesting potential termination defects in this strain. Previous work has demonstrated significant read-through of transcriptional terminators by *rpa12Δ* Pol I [19]. To characterize the effect of *rpa12Δ* on termination using NET-seq experiments, we examined the median polymerase occupancy both upstream and downstream of T1 and T2 (Figure 5A). As expected, we observed increased WT Pol I occupancy directly upstream of T1 followed by a significant decrease directly downstream. Very little signal for WT Pol I occupancy is detected adjacent to the T2 termination site (Figure 5A). These patterns are consistent with the initial transcriptional pause required for termination of Pol I transcription and highly efficient termination at T1. In contrast, we do not see any clustering of *rpa12Δ* Pol I adjacent to either terminator site, indicating that *rpa12Δ* Pol I does not pause at these sites like WT Pol I (Figure 5A). Together, these data suggest that unlike WT Pol I, *rpa12Δ* Pol I is not reliably terminating at the designated transcription termination sites, and instead is still engaged with the template well beyond both T1 and even T2. 

We also observed persistent *rpa12Δ* Pol I occupancy throughout IGS 1, the 5S gene, and IGS 2 (Figure 5B). To detect signal via NET-seq, the EC must be associated with a nascent transcript, indicating that these polymerases are actively transcribing. How do these polymerases escape Rat1-dependent termination? These data suggest that the elongation rate of *rpa12Δ* Pol I is greater than the processive nucleotide excision rate of Rat1. Alternatively, Rat1 binding to the nascent transcript may rely on transcriptional pausing at T1 or assembly of precise RNA structures that may be perturbed in the mutant strain. Interestingly, WT Pol I signal was also observed downstream of T2 at one distinct locus, which was directly upstream (relative to Pol I transcription) of the 5S gene. In fact, this is the only location in the IGS where Pol I occupancy is greater in the WT strain than the *rpa12Δ* strain. These data show that a very small subset of WT Pol I ECs read through both the primary termination site (T1) and the secondary fail-safe site (T2), with the ECs ultimately halting upon encountering the 3′ end of the 5S gene, likely due to collision with Pol III or its associated factors. By contrast, *rpa12Δ* results in significantly increased Pol I EC read-through of T1 and T2, as well as the 5S gene, suggesting that this deletion renders the polymerases termination deficient. 

Our lab has identified that the *rpa12Δ* Pol I EC is much more stable than WT in vitro [24], and this additional stability likely allows *rpa12Δ* Pol I ECs to read through the Pol III EC-occupied 5S gene. Compared to WT, we see consistent *rpa12Δ* Pol I occupancy throughout IGS 2, culminating in a cluster of positions with sharply increased occupancy approximately 215 base pairs upstream of the transcription start site for the adjacent 35S gene (Figure 5B). Interestingly, this site is also just upstream of the promoter-proximal binding site for Reb1 (Figure 5B). These data suggest that this position represents a third, previously uncharacterized, site for Pol I EC pausing/arrest and possibly transcription termination. This site could be crucial to ensure that termination occurs prior to read-through into the next rDNA promoter. Taken together, there is robust occupancy of Pol I ECs in the IGS regions in the *rpa12Δ* strain. This read-through could have many negative consequences, such as induction of DNA damage and disruption of 5S rRNA synthesis.

## 4. Discussion

### 4.1. Pol I Occupancy Patterns within the 35S Gene Change in Response to RPA12 Deletion

Initial characterization of the mechanistic contribution of A12.2 to Pol I function focused on transcript cleavage and termination [18,19,20]. However, recent studies of Pol I nucleotide addition in vitro show that removal of the A12.2 subunit from the Pol I EC changes the kinetics of nucleotide incorporation [24,25,29]. This observation indicates that A12.2 plays a role in transcription elongation as well as termination and transcript cleavage. Analysis of our NET-seq data corroborates these findings in vivo. NET-seq revealed substantial shifts in *rpa12Δ* Pol I occupancy throughout the 35S gene, including increased occupancy in the 5′ end of the gene relative to WT (Figure 4). By preparing these libraries in biological triplicate, we determined that many of these changes in occupancy were statistically significant (Figure 4). These data are consistent with transcription run-on data and Miller chromatin spreads published by the Beyer and Proudfoot labs [19], indicating a 5′ bias in *rpa12Δ* Pol I EC distribution. The lack of cleavage activity by the mutant Pol I may play a role in this observed 5′ occupancy shift. Reductions in TFIIS-mediated transcript cleavage by Pol II have been shown to impair yeast viability and Pol II transcription elongation in vivo [41]. The permanent integration of the TFIIS paralogue A12.2 into Pol I suggests that cleavage activity is important for the proper function of Pol I. Deletion of *RPA12* induces a two-fold decrease in the growth rate of yeast and considering the transcription effects found in the *rpa12Δ* strain, this mutation will likely contribute to defects in rRNA processing. Pol I ECs are also very densely packed on each active rDNA repeat, often exceeding 50 ECs per 35S gene [1]. Thus, stable *rpa12Δ* polymerase stalling on the rDNA template (coupled with the increased stability of the *rpa12Δ* Pol I EC [24,42]) may represent a substantial barrier for Pol I ECs in the 5’ end of the gene. This accumulation of “roadblocks” might explain the observed accumulation of *rpa12Δ* Pol I ECs in the 5′ end of the 35S gene. Consistent with this model, previous studies of head-to-tail collision events between Pol II ECs have indicated that collision with the leading polymerase causes the trailing polymerase to backtrack in vitro, relying on TFIIS-mediated cleavage to continue productive elongation [43]. The resolution of such backtracked complexes for Pol I is impaired in *rpa12Δ* cells. 

The template positions demonstrating statistically significant changes in polymerase occupancy displayed unique sequence trends within the RNA:DNA hybrid, and overall sequence-specific repositioning (Figure 2). We identified repositioning of *rpa12Δ* Pol I occupancy on the DNA template in the top 2.5% occupied positions. The cumulative effect of this may explain the observed 5′ preference in occupancy in the *rpa12Δ* strain as well as the significant increase in spacer region occupancy. Overall, these findings show that nucleotide addition by *rpa12Δ* Pol I is perturbed in vivo, consistent with previous in vitro studies [25,26,29]. They also provide a potential mechanistic explanation for the in vitro findings. Additional mutational analysis would further elucidate the role that the A12.2 subunit plays in nucleotide addition. For example, the C-terminal domain (CTD) of A12.2 is responsible for conferring intrinsic cleavage activity to Pol I [18], whereas the N-terminal domain is responsible for anchoring A12.2 within Pol I [20]. By analyzing Pol I occupancy in A12.2 CTD deletion yeast strains, the specific contributions of the transcript cleavage and anchoring domains to the perturbations in Pol I occupancy observed could be determined. 

### 4.2. S. cerevisiae Contains a Putative Third Pol I Terminator Region

Our NET-seq data confirm that deletion of *RPA12* renders Pol I termination-deficient in vivo, as we observed significant *rpa12Δ* Pol I EC read-through of both previously defined sites of Pol I termination (Figure 5A). Furthermore, we observed increased *rpa12Δ* Pol I occupancy throughout the IGS compared to WT (Figure 5B). Interestingly, *rpa12Δ* Pol I occupancy was enriched at a site directly upstream of the promoter-proximal Reb1 binding site, indicating *rpa12Δ* Pol I is pausing or arresting at this position. Reb1 has a higher affinity for this site than its terminator-proximal binding site and appears capable of halting *rpa12Δ* Pol I, whereas Nsi1 binding to the T1-proximal site is not (Figure 5B) [10]. As NET-seq is not sensitive to termination events due to it only reporting on nascent RNA, we cannot decisively conclude that this site induces termination of Pol I transcription. However, Pol I transcription termination involves pausing the EC as the first step [44]. These data raise the question of whether this site represents an even stronger Pol I terminator than T1. This third site could function as a final failsafe, preventing the collision of Pol I ECs and the transcription initiation machinery bound to the promoter region of the adjacent 35S repeat. This arrangement has been observed in higher eukaryotes such as *Xenopus laevis* [45] and mice [46]. However, our data suggest that WT Pol I does not reach this site in vivo under normal conditions. 

Why then has this site been preserved if it is unnecessary under normal growth conditions? In the WT strain, a population of polymerases read through both T1 and T2, resulting in Pol I occupancy near the 3′ end of the 5S gene (Figure 5B). Under the growth conditions tested, we did not observe consistent WT signal beyond the 5S gene, suggesting that the WT Pol I EC cannot transcribe past actively transcribed Pol III repeats. It is reasonable to suggest that Pol III ECs on the 5S gene interfere with Pol I transcription, resulting in Pol I transcriptional arrest. Perhaps in slower growth conditions or during stress when ribosome synthesis is reduced, Pol III loading on the 5S gene would be decreased, increasing the probability of Pol I EC read-through into IGS 2. Under such conditions, a promoter-proximal terminator site would prevent Pol I EC collisions with transcription initiation factors at the next 35S gene. 

In addition to NET-seq, many other high-throughput techniques have recently been developed and utilized to capture a global perspective of the positioning of RNA polymerases on a DNA template. Many of these alternative techniques use a similar protocol to that in this study, with one major difference being the introduction of crosslinking to purify elongation complexes. A recent publication demonstrates the use of the crosslinking and cDNA analysis (CRAC) method to probe for Pol I occupancy in WT yeast [47]. In agreement with our WT NET-seq data included here, as well as previous results from our lab [27,28,29], the CRAC data show that the distribution of Pol I is heterogeneous across the 35S gene. However, there are some differences in the occupancy patterns shown in the NET-seq versus CRAC datasets. This could be due to an unintended effect of the crosslinking step in the CRAC technique, as NET-seq is not designed to detect indirect transient interactions. Alternatively, the lack of crosslinking in NET-seq may result in the loss of signal due to collapsed elongation complexes. This observation seems unlikely, given the demonstrably long half-life of elongation complexes in vitro, but such an effect is possible. Importantly, this study deployed identical experimental strategies to test the effects of deletion of *RPA12*. The results corroborate previous studies and highlight the extensive roles for A12.2 in transcription elongation and termination in vivo. 

Analysis of Pol I occupancy on the rDNA via NET-seq refines our understanding of the role for A12.2 in Pol I function in vivo, as well as the architecture of the yeast IGS. These data also suggest several new lines of inquiry. How does A12.2 moderate Pol I occupancy in a sequence-dependent manner? Which domain of A12.2 is responsible for this behavior? Does the promoter-proximal binding site represent a third site of Pol I termination and, if so, what purpose does it serve in *S. cerevisiae*? How well-conserved are these termination sites in other eukaryotic species? Answering these questions will bring us closer to a fundamental understanding of Pol I, and the genomic region it transcribes. These high-resolution in vivo findings demonstrate that the A12.2 subunit of Pol I plays an important role in transcription elongation and termination. Furthermore, these data suggest that there may be a third, previously uncharacterized, transcription termination site for Pol I in yeast.

## 5. Conclusions

NET-seq analyses demonstrate that the A12.2 subunit of Pol I plays an important role in transcription elongation and termination in living cells, consistent with previous findings in vitro. Furthermore, these data suggest that there may be a third, previously uncharacterized, transcription termination site for Pol I in yeast.

## Data Availability

Raw data files can be accessed via the Gene Expression Omnibus database with the accession number GSE142457.
